# Transient Pulmonary Artery Hypertension in Holstein Neonate Calves

**DOI:** 10.3390/ani10122277

**Published:** 2020-12-03

**Authors:** Melina Marie Yasuoka, Bruno Moura Monteiro, Paulo Fantinato-Neto, Renan Braga Paiano, Denise Tabacchi Fantoni, Denise Aya Otsuki, Eduardo Harry Birgel Junior

**Affiliations:** 1 Department of Veterinary Medicine, College of Animal Science and Food Engineering, University of São Paulo, Pirassununga 13635900, Brazil; fantinatont@gmail.com (P.F.-N.); ehbirgel@usp.br (E.H.B.J.); 2 Department of Animal Reproduction, School of Veterinary Medicine and Animal Sciences, University of São Paulo, São Paulo 05508270, Brazil; brunomouramonteiro@hotmail.com; 3 Department of Surgery, Faculty of Veterinary Medicine and Animal Science, São Paulo University, São Paulo 05508270, Brazil; dfantoni@usp.br; 4 LIM-08, School of Medicine, University of São Paulo, São Paulo 01246000, Brazil; deniseotsuki@hotmail.com

**Keywords:** blood gas, neonates calves, cardiac output, pulmonary artery pressure

## Abstract

**Simple Summary:**

At birth, calves are challenged to maintain their vital functions that were previously supported by the placenta of cows. Failures in the neonatal adaptation process can occur during this and can cause the death of neonates. The present study aimed at the invasive hemodynamic evaluation of calves during the first 30 days of life to elucidate the changes in calves during this stage of life. In conclusion, the results of this research show a transient pulmonary arterial hypertension during the process of adapting to extrauterine life.

**Abstract:**

The neonatal period is a challenging phase for calves, and during this phase constant adaptations are required. The aim of the present study was to evaluate the invasive hemodynamics with the Swan-Ganz catheter in neonate calves to understand adaptive changes during the first 30 days of life. A prospective and observational study was conducted with 10 Holstein calves. Assessments of the right atrial pressure (RAP), right ventricular pressure (RVP), pulmonary artery pressure (PAP), pulmonary capillary pressure (PW), cardiac output (CO), heart rate (HR), pulmonary vascular resistance (PVR), and blood gas levels were performed. The analyses of PAP, PVR, PW, HR, sO_2_, and arterial blood gases differed (*p* < 0.05) between the evaluated periods. Our results indicated transient pulmonary artery hypertension during the process of extrauterine adaptation during the first 30 days of life. This hypertension must be considered as physiological and consequent to the neonatal adaptation process.

## 1. Introduction

Parturition is a challenging period for both the cow and the newborn calf [1,2]. During this phase profound physiological changes occur [3,4,5], and adaptations to the extrauterine environment are essential for the calf’s vitality and health [6]. Among these adaptations, particular emphasis needs to be placed on those that are performed by the cardiorespiratory system that supports the profound changes that occur in blood circulation, such as the closure of the ductus arteriosus that is caused by increased blood oxygenation. The inflation of the lungs, which causes a decrease in resistance in the pulmonary capillary vessels, increases blood flow to the lungs, and a higher volume of blood returns to the left atrium with a resulting increase in pressure in this location, which in turn induces the oval foramen closure, thereby preventing the mixing of venous and arterial blood [7].

Failures in the process of neonatal adaptation are often associated with high rates of morbidity and mortality in calves within the first month of life [8]. Among the main causes of death of calves in the first days of life, we can highlight early or late neonatal asphyxia [9]. Due to disturbances in oxygenation that are associated with neonatal asphyxia, the following symptoms are observed: a decreased partial pressure of oxygen (pO_2_), an increased partial pressure of carbonic dioxide (pCO_2_), preferential circulation to the vital organs, and metabolic acidosis [10]. It has been reported that in response to these clinical conditions, pulmonary hypertension could develop owing to hypoxia and persistent vasoconstriction and may even result in the reversal of pulmonary blood flow in a direction that is opposite to the pattern that is observed during fetal life with the opening of the foramen ovale and the presence of the patent ductus arteriosus [7].

Pulmonary hypertension is a pathological condition that is observed in association with heart diseases, systemic diseases, and the diseases of the pulmonary parenchyma or pulmonary vasculature that determine the development of pulmonary endothelium dysfunction, which is expressed by vasoconstriction, inflammation, and thrombosis [11]. Among the many factors that are related to the occurrence of pulmonary hypertension in calves, meconium aspiration that may occur during birth should be highlighted [7]. The development of cardiorespiratory problems, particularly of pulmonary hypertension that causes the persistence of the ductus arteriosus and foramen ovale, seems to be the most influential factor in the survival of the newborn premature calf [12].

Pulmonary hypertension is an issue that requires further studies into the cattle’s neonatology. The lack of information on pulmonary hypertension in newborn calves makes it difficult to diagnose correctly and to start the early administration of therapeutic protocols. The Swan-Ganz catheter allows the diagnosis of pulmonary hypertension in the first days of the newborn. The technique also allows for the measurement of the pressures in the right atrium and ventricle, pulmonary artery pressure, pulmonary capillary wedge pressure, and the cardiac output via the thermodilution and blood gas analysis of the cardiac chambers [13,14]. Therefore, the aim of this research was to perform invasive hemodynamic monitoring with the Swan-Ganz catheter in non-sedated calves, in order to determine the pulmonary capillary wedge pressure (PW), cardiac output (CO), heart rate (HR), pulmonary vascular resistances (PVR), and blood gas levels of the cardiac chambers to understand the modifications in newborn calves in the first 30 days of life.

## 2. Materials and Methods

### 2.1. Ethical Statement

All animal procedures were approved by the Bioethics Committee of the School of Veterinary Medicine and Animal Sciences, University of São Paulo, São Paulo, Brazil (1927/2010).

### 2.2. Animals

The study was conducted at the School of Animal Husbandry and Food Engineering at the University of São Paulo, Pirassununga, Brazil. In total, ten newborn male Holstein calves that were conceived by artificial insemination and birthed via natural births were used in this study. The calves arrived at the school and received colostrum while they waited for the experimental procedures. During the experimental period, the animals were bottle-fed with four liters of milk, twice a day, and they had free access to hay and water.

### 2.3. Sample Collection and Analysis

The samples were collected at the following time points: 1st, 7th, 15th, and 30th days of life of the calves. Analyses of the HRs, O_2_ saturation (sO_2_), RAP, RVP, PAP, PW, CO, PVR and blood gas levels in the vena cava, right atrium and ventricle, and the pulmonary artery (mixed venous blood) were performed.

Prior to catheter insertion, the area was infiltrated with 1mL of 2% lidocaine. An introducer that was set at 8.5F (Edwards Life Sciences Ltd.a) was placed into the right jugular vein, following which a 110 cm 7F Swan-Ganz thermodilution catheter was introduced aseptically into the vein and connected to a fluid-filled transducer (PX260, BD). The transducer was at the heart level and was used as a zero-reference level.

Characteristic tracing patterns that were observed on a monitor were used to confirm correct catheter positioning. The catheter was permanently flushed with heparinized 0.9% sterile saline solution, and in order to measure the blood pressure, the transducer was connected to a monitor (DASH 3000- GE). The RAP, RVP, PAP, and PW were measured via the different ports of the Swan-Ganz catheter. Intracardiac pressure measurements were performed after the stabilization parameter was determined, and following this assessment, blood samples were collected for a hemogasometric evaluation.

CO was calculated using the thermodilution technique by injecting 10 mL of cold (0–5 °C) 5% dextrose through the proximal port of the catheter, which was repeated three times to obtain a mean value [14]. Following the baseline measurements that were performed while the animals were in left lateral recumbency, they were free to stand up.

The pulmonary vascular resistance (PVR) was calculated according to the following formula [15], the result being expressed in dyna:

PVR = (PAP) − (PaPo)/(Cardiac Output) × 80

### 2.4. Statistical Analysis

The data were analyzed using repeated measures of analysis of variance (ANOVA) by the MIXED procedure of SAS (SAS Institute Inc., Cary, NC, USA, Version 9.3) for multiple comparisons of the means by LSMEANS (Least Squares Means), and Tukey’s test adjustment. Data were assessed for normality using the Shapiro–Wilk test. Variables without normal distribution were analyzed for the time effect using the Friedman test and analyzed comparing at each time point using Wilcoxon’s test (SAS Institute Inc., Cary, NC, USA, Version 9.3), with *p* < 0.05 considered to be statistically significant. Data are presented as means and ± standard errors of the means (SEM).

## 3. Results

The pressure curves were stable, and the variations observed in values associated with the measurements were insignificant. Via the measurements performed on day 1, it was found that 10% (1/10) of the calves presented a higher pressure on the right side of the heart compared to on the left side. Additionally, 20% (2/10) of the calves presented equal pressures on both sides of their hearts. Following the first 7 days of life, this was not observed via any of the 30 measurements that were performed.

**Figure 1:**
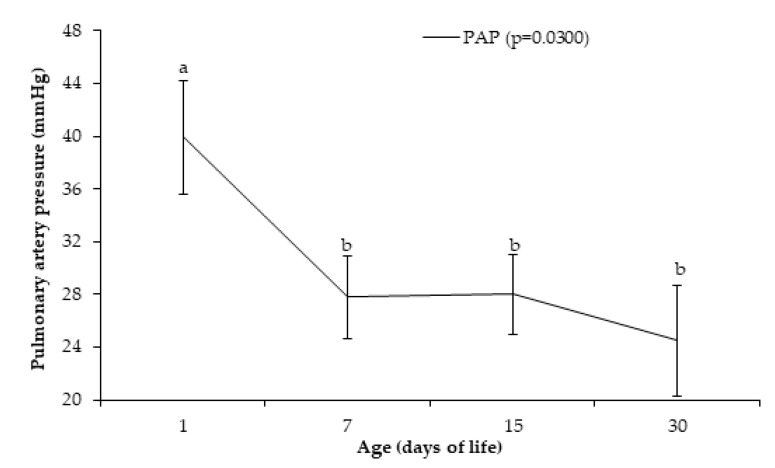
Pulmonary artery pressure -PAP (means ± SEM) of Holstein calves at 1, 7, 15, and 30 days after birth. ^a,b^ different lowercase letters indicate differences between ages.

The PAP (Figure 1) that was observed in the newborns examined on day 1 following birth (39.90 ± 4.28 mmHg) was higher (*p* = 0.030) than the values observed on days 7 (27.80 ± 3.16 mmHg), 15 (28.00 ± 3.01 mmHg), and 30 (24.50 ± 4.19 mm Hg).

**Figure 2:**
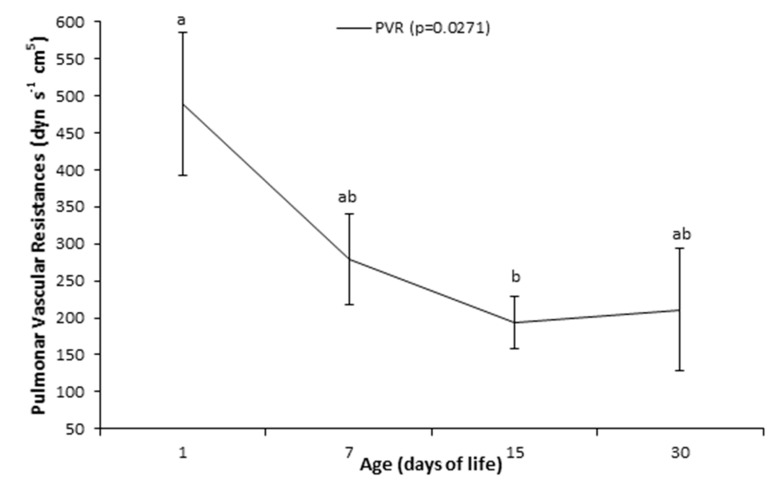
Pulmonary vascular resistances -PVR (means ± SEM) of Holstein calves at 1, 7, 15, and 30 days after birth. ^a,b^ different lowercase letters indicate differences between ages.

The PVR (Figure 2) was higher (*p* = 0.027) on day 1 (488.92 ± 97.11 dyn s^−1^ cm^5^) than on day 15 (194.14 ± 35.68 dyn s^−1^ cm^5^).

**Figure 3:**
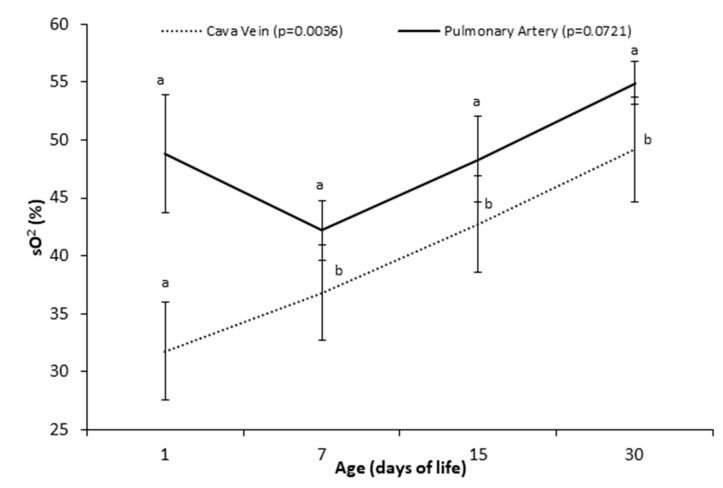
Oxygen saturation of vena cava/pulmonary artery blood (means ± SEM) of Holstein calves at 1, 7, 15, and 30 days after birth. ^a,b^ different lowercase letters indicate differences between ages.

No difference was observed for the sO_2_ of the pulmonary artery (Figure 3), while the sO_2_ in cava vein in the blood samples collected on the 1st day was lower (*p* = 0.003) than on days 7, 15, and 30.

The results of RVP, RAP, pulmonary capillary wedge pressure, CO, and HR are presented in Table 1. The PVR was higher (*p* = 0.027) on day 1 than on day 15. No difference was observed for the right atrium pressure, while for the pulmonary capillary wedge pressure, the result on day 1 was lower (*p* = 0.013) than on days 7 and 15. No difference was observed for cardiac output. The HR recorded on day 1 following birth were higher (*p* = 0.0002) than those obtained after days 7 and 15.

Complementing the values obtained via the Swan-Ganz catheter, blood gas analysis was performed in the vena cava, right atrium, right ventricle, and pulmonary artery (mixed venous blood), the results of which are presented in Table 2. The values of pH (for vena cava, right atrium, right ventricle, and pulmonary artery), pO_2_ (for vena cava and right atrium), pCO_2_ (for pulmonary artery), bicarbonate (for vena cava, right atrium, right ventricle, and pulmonary artery) and BE (for vena cava, right atrium, right ventricle and pulmonary artery) differed (*p* < 0.05) between the periods evaluated. The pH values on the first day for vena cava, right atrium, right ventricle, and pulmonary artery were lower (*p* < 0.05) than those observed on day 30. The pO_2_ value for vena cava was lower (*p* < 0.05) on the day 1 than on day 30, while for the right atrium, the value on day 1 was lower (*p* < 0.05) than that of days 15 and 30, and of day 15 was lower (*p* < 0.05) than that noticed on day 30. Regarding pCO_2_, the value for the pulmonary artery was lower (*p* < 0.05) on day 1 than on day 30. Bicarbonate values were lower (*p* < 0.05) on days 1 and 7 than on day 30 for vena cava, in the right atrium the value of day 15 was lower (*p* < 0.05) than that of day 30, in the right ventricle the values of days 1, 7, and 15 were lower (*p* < 0.05) than that observed at day 30, while in the pulmonary artery the values of days 1 and 7 were lower (*p* < 0.05) than that of day 30. According to the BE results, for the vena cava the values of days 1 and 7 were lower (*p* < 0.05) than those of days 15 and 30, for the right atrium the value of day 15 was lower (*p* < 0.05) than that of day 30, for the right ventricle and pulmonary artery the values of days 1 and 7 were lower (*p* < 0.05) than what was noticed on day 30.

## 4. Discussion

Pulmonary hypertension is a disorder that needs to be better studied in newborn calves. The main factors associated with the occurrence of pulmonary hypertension in calves are meconium aspiration during calving, cases of persistent hypoxia caused by pneumonia, and factors related to animals raised in high altitude conditions [5]. Pulmonary hypertension has been reported in calves from biotechnology procedures, such as cloning from the nuclear transfer of adult somatic cells [16,17].

The increase in resistance in the pulmonary capillary network, determined by pulmonary artery hypertension, prevents adequate pulmonary expansion and is associated with respiratory changes in neonates [18]. The pulmonary insufflation depends on the neonate’s first inspiration; thus, higher lung capacity can contribute to an increase in the neonate’s survival rate [19]. The results of this research demonstrate the occurrence of transient pulmonary artery hypertension that developed during the process of adaptation to extrauterine life. This hypertension must be considered as physiological and as a consequence of the neonatal adaptation process. By inflating the lungs in the first moments after birth, the neonate decreases the resistance in the pulmonary capillary network, which in turn leads to an increase in the blood flow to the lungs, increasing the volume of blood resulting from the recovery of the left atrium and, consequently, the pressure on the left side of the heart increases [7]. The development of pulmonary hypertension observed in newborn calves implies, as would be expected, that lung expansion does not occur completely in the first moments of life, and it may be an indication of the closure of the foramen ovale and the ductus arteriosus, which is a process that occurs during the 1st days of life. As can be seen in Table 1, the observed pulmonary vascular resistance decreases, with the value found being higher on the first day of life than in the 15th day of life. Another cause of transient pulmonary hypertension previously described in calves is related to the contact between the tip of the Swan-Ganz catheter and the arterial wall, which can lead to a reaction of local mechanical sensors contributing to the occurrence of pulmonary hypertension [20].

According to the PAP values, Amory et al. [21] have suggested that the PAP in calves decreases rapidly from birth until 12 h following birth and continues decreasing until it reaches a plateau; during the period before the calves are 1 week old and from when they are in their first week until they are 1 year old. In agreement with that, we found a high PAP during the first day of life in comparison with measurements performed on the other days. Values of PAP above 40 mmHg can be characterized as pulmonary artery hypertension in newborn calves [20]. Values lower than those observed in the present study were previously described [22]. Anesthesia induction does not decrease the PAP values and can occasionally increase the PAP [23]. Neary et al. [24] reported higher PAP values associated with 1-month-old male calves located at high altitudes (greater than 2000 m). Other authors have compared PAP values between animals that were born and raised at high altitudes and those born at low altitudes [25], and it appears that altitudes above 1500 m are strongly related to higher PAP values. However, our study was conducted at an altitude of 622 m, which is a relativity low altitude to cause any interference with pulmonary physiology.

Regarding PVR, RVP, PW, and sO_2_, values similar to ours were observed in newborn calves in a study carried out in Switzerland [26], Belgium [27] and the United States [28].

The decrease in the pressure in the right ventricle was accompanied by a decrease in PAP and an increase in PW, facilitating the process of adaptation to extrauterine life, during which the pressure on the left side of the heart increases with age. Improved lung capacity results in decreased vascular resistance and a reduction in the pulmonary artery and right ventricular pressures. These pressure variations are essential for the foramen ovale and ductus arteriosus closures that prevent the deoxygenated blood from mixing with the oxygenated blood, which could lead to significant hypoxia and could compromise the viability of the neonate.

The evaluation of sO_2_, when compared with blood samples from the cranial vena cava and pulmonary artery, can be an important method for the diagnosis of left-right cardiac shunt. The occurrence of intracardiac shunts can lead to changes in blood sO_2_ [29]. If the difference in sO_2_ is 8% or more between these samples, a left-to-right “shunt” may be present [29]. Based on our results, we observed a difference of 17.03%, 5.4%, 5.63%, and 5.7% on days 1, 7, 15, and 30, respectively. Thus, the difference observed on day 1 was greater than 8%, suggesting the presence of a shunt from left to right [30]. Possibly, in the first 24 h of life, the studied calves had a patent foramen ovale, the closure of which may not have occurred until the 7th day after birth [30]. From the 7th until the 30th day, the differences in saturation did not exceed 8%.

Previous studies have shown that left ventricular output increases twofold to threefold compared to fetal left ventricular output after birth [31]. The main regulator of left ventricular output immediately after birth is the stroke volume and not heart rate [32]. Pulmonary blood flow increased approximately six-fold after the beginning of breathing and interruption of placental circulation and directly resulted in an increase in left ventricular preload [33]. In addition, the ductus arteriosus remained widely patent shortly after birth. This left-to-right shunt probably plays a role in increasing left ventricular output at 1 h of age [30]. In human newborns, at 24 h of age, the left ventricular output decreases to 75% of the value of 1 h and then remains unchanged until 96 h of life [32]. In the present study, it was demonstrated that the cardiac output of the right ventricle was constant from the first to the 30th day of life. We did not find information about changes in cardiac output immediately after birth for newborn calves. Pogliani [30] observed, by echocardiography, that the cardiac output of Nellore calves in the first month of life was not influenced by age, with values ranging between 4.2 ± 1.1 and 5.3 ± 1 8 L/min. These cardiac output values are similar to those found in the present study.

Based on the results of the HR, the values described in the present study are in agreement with those previously reported [27]. We highlight that after day 15, the HR of the animals evaluated stabilizes. Pogliani [30], evaluating the HR of Nellore calves obtained both by natural mating and by nuclear transfer of somatic cells in Brazil, reported that HR decreased after the first days of life. According to Feitosa et al. [34], the decrease in HR of calves occurs mainly during the first 24 h of life and may decrease from 143.72 ± 21.45 beats/min (immediately after birth) to 116.72 ± 17.68 beats/min (24 h after birth). If the heart rate decreases and the cardiac output remains the same, there will necessarily be an increase in stroke volume with advancing age. The amount of blood ejected from the left ventricle observed in the first 30 days of life for Nellore calves increased from 57.4 ± 7.1 mL to 92.5 ± 11.1 mL [30]. As the newborn’s organism is not yet able to vary cardiac output, it compensates for the limited systolic volume by the increase in heart rate [35]. This may explain the effect of days of life during the first month on decreasing calves’ heart rate. Due to the newborn’s dependence on heart rate for cardiac output, bradycardia can result in decreased blood pressure and eventual cardiovascular collapse, so low or falling heart rates require immediate attention.

The observed values for the pH, bicarbonate, and BE are in accordance with those described as physiological for newborn calves [36]. Newborn calves show less oxygenation on day 1, which leads to a lower pH. However, the pH values observed in the present study are compatible with life since they are within the normal range [36]. Immediately after birth, newborns may have an immature pulmonary system, and their maturation may occur a few months or years after birth, depending on the species [37,38]. A possible explanation for maintaining pH is a higher consumption of BE and bicarbonate. Thus, the improvement in oxygenation that occurs over the 30th day after birth is reflected by a slight increase in pH and a decrease in the consumption of BE and bicarbonate. We also highlight that the increase in pH, pO_2_ and sO_2_, BE, and bicarbonate values may have been influenced by age. Similar results were observed by Vannucchi et al. [6] in a study carried out on neonatal Holstein calves submitted to different calving times and obstetric assistance.

## 5. Conclusions

In conclusion, although this study was carried out with a small number of animals, our data show that the use of the Swan-Ganz catheter in newborn calves that are not sedated is safe, including in emergency practice, and the resulting data allow for the diagnosis of changes in neonatal adaptation, facilitating the course of treatment to be tailored to the specific animal. There is no need for the sedation of the animals or the induction of anesthesia prior to obtaining excellent pressure curves. The results demonstrate the feasibility of the technique, and owing to the analysis of the inherent risks associated with its use, we can state that this technique that involves the invasive monitoring of the right side of the heart with a Swan-Ganz catheter may be used without restriction in order to diagnose respiratory diseases and in experimental protocols, including those that are aimed at evaluating the effects of various drugs on PAP. In addition, we highlight that the results of the present longitudinal study show transient pulmonary arterial hypertension during the first month of life in newborn calves. This hypertension must be considered physiological and consequent to the process of neonatal adaptation to extrauterine life. Therefore, as it is considered a physiological adaptation, we do not recommend any therapeutic protocol for this type of transient pulmonary hypertension.

## Figures and Tables

**Table 1:** Means ± SEM of hemodynamic parameters of Holstein calves at 1, 7, 15, and 30 days after birth.

Variable	Age (Days of Life)			
	1st Day	7th Day	15th Day	30th Day
Right ventricle pressure (mmHg)	29.20 ± 2.44 ^a^	22.50 ± 1.41 ^b^	20.90 ± 1.40 ^b^	18.50 ± 1.55 ^b^
Right atrium capillary wedge pressure (mmHg)	4.80 ± 0.96	6.00 ± 0.99	5.10 ± 0.57	5.10 ± 0.81
Pulmonary capillary wedge pressure (mmHg)	7.20 ± 1.02 ^b^	10.10 ± 0.95 ^a^	12.10 ± 0.80 ^a^	9.30 ± 1.19 ^ab^
Cardiac output (L/min)	5.86 ± 0.55	5.97 ± 0.69	7.01 ± 0.66	6.73 ± 0.47
Heart rate (bpm)	126.10 ± 5.44 ^a^	110.30 ± 4.87 ^b^	98.60 ± 2.20 ^bc^	99.10 ± 4.67 ^c^

^a,b,c^ Values within a row (across evaluation times) with different superscripts differ (*p* < 0.05).

**Table 2:** Blood gas values (means ± SEM) of Holstein calves at 1, 7, 15, and 30 days after birth.

Variable ^1^	Sample Source	Age (Days of Life)			
		1st Day	7th Day	15th Day	30th Day
pH	Vena cava	7.351 ± 0.01 ^b^	7.358 ± 0.02 ^ab^	7.374 ± 0.01 ^ab^	7.403 ± 0.01 ^a^
	Right atrium	7.353 ± 0.01 ^b^	7.356 ± 0.01 ^b^	7.362 ± 0.01 ^b^	7.414 ± 0.01 ^a^
	Right ventricle	7.359 ± 0.01 ^b^	7.365 ± 0.01 ^b^	7.372 ± 0.01 ^ab^	7.408 ± 0.01 ^a^
	Pulmonary artery	7.362 ± 0.01 ^b^	7.369 ± 0.01 ^b^	7.383 ± 0.01 ^ab^	7.406 ± 0.01 ^a^
pO_2_ (mmHg)	Vena cava	23.20 ± 1.67 ^b^	25.60 ± 1.87 ^ab^	29.70 ± 1.48 ^a^	30.60 ± 2.14 ^a^
	Right atrium	25.10 ± 2.10 ^c^	28.60 ± 1.19 ^bc^	30.60 ± 1.75 ^b^	35.40 ± 0.90 ^a^
	Right ventricle	27.20 ± 2.00	29.00 ± 0.89	28.50 ± 1.93	29.90 ± 1.86
	Pulmonary artery	30.40 ± 2.37	28.50 ± 0.970	32.66 ± 1.76	33.00 ± 0.96
pCO_2_ (mmHg)	Vena cava	52.64 ± 1.95	48.76 ± 1.89	52.60 ± 1.91	52.26 ± 1.00
	Right atrium	49.71 ± 2.75	48.30 ± 1.50	47.43 ± 3.17	49.68 ± 0.74
	Right ventricle	47.89 ± 2.19	47.27 ± 1.67	48.88 ± 2.40	52.33 ± 1.30
	Pulmonary artery	50.32 ± 0.80 ^ab^	46.85 ± 1.64 ^b^	49.78 ±1.56 ^ab^	51.23 ± 0.94 ^a^
Bicarbonate (mmol/L)	Vena cava	28.97 ± 1.18 ^b^	27.53 ± 1.59 ^b^	30.47 ±1.04 ^ab^	32.33 ± 0.60 ^a^
	Right atrium	28.49 ± 1.13 ^ab^	27.01 ±1.38 ^ab^	27.31 ± 2.30 ^b^	31.52 ± 0.75 ^a^
	Right ventricle	27.92 ± 0.85 ^b^	26.96 ± 1.41 ^b^	29.62 ± 1.35 ^b^	32.82 ± 0.77 ^a^
	Pulmonary artery	28.53 ± 1.00 ^b^	26.93 ± 1.45 ^b^	30.50 ±1.23 ^ab^	35.04 ± 2.94 ^a^
BE (mmol/L)	Vena cava	3.90 ± 1.34 ^b^	2.60 ± 1.92 ^b^	5.80 ± 1.26 ^a^	8.10 ± 0.75 ^a^
	Right atrium	3.30 ± 1.31 ^ab^	2.00 ± 1.67 ^ab^	2.40 ± 2.55 ^b^	7.20 ± 0.95 ^a^
	Right ventricle	2.80 ± 1.08 ^b^	2.20 ± 1.57 ^b^	5.70 ± 1.66 ^ab^	8.60 ± 0.90 ^a^
	Pulmonary artery	3.40 ± 1.24 ^b^	2.30 ± 1.71 ^b^	5.88 ± 1.44 ^ab^	7.70 ± 0.92 ^a^

^a,b,c^ Values within a row (across evaluation times) with different superscripts differ (*p* < 0.05). ^1^ pO_2_ = partial pressure of O_2_; pCO_2_ = partial pressure of CO_2_; BE = base excess.

## References

[1] Paiano R.B., Birgel D.B., Birgel Junior E.H. (2020). Influence of peripartum on the erythrogram of Holstein dairy cows. J. S. Afr. Vet. Assoc..

[2] Paiano R.B., Birgel D.B., Bonilla J., Birgel Junior E.H. (2020). Evaluation of biochemical profile of dairy cows with metabolic diseases in tropical conditions. Reprod. Domest. Anim..

[3] Paiano R.B., Lahr F.C., Silva L.S.B., Marques D.S., Ferreira C.A., Birgel D.B., Bisinotto R.S., Birgel Junior E.H. (2019). Haematological and biochemical profiles during the puerperium in dairy cows. Acta Vet. Hung..

[4] Paiano R.B., Birgel D.B., Ollhoff R.D., Birgel Junior E.H. (2019). Biochemical profile and productive performance in dairy cows with lameness during postpartum period. Acta Sci. Vet..

[5] Paiano R.B., Birgel D.B., Bonilla J., Birgel Junior E.H. (2020). Alterations in biochemical profiles and reproduction performance in postpartum dairy cows with metritis. Reprod. Domest. Anim..

[6] Vannucchi C.I., Silva L.G., Lúcio C.F., Veiga G.A.L. (2019). Oxidative stress and acid–base balance during the transition period of neonatal Holstein calves submitted to different calving times and obstetric assistance. J. Dairy Sci..

[7] Poulsen K.P., McGuirk S.M. (2009). Respiratory disease of the bovine neonate. Vet. Clin. N. Am. Food Anim. Pract..

[8] Dillane P., Krump L., Kennedy A., Sayers R.G., Sayers G.P. (2018). Establishing blood gas ranges in healthy bovine neonates differentiated by age, sex, and breed type. J. Dairy Sci..

[9] Murray C.F., Leslie K.E. (2013). Newborn calf vitality: Risk factors, characteristics, assessment, resulting outcomes and strategies for improvement. Vet. J..

[10] Benesi F.J. (1993). Síndrome asfixia neonatal dos bezerros. Importância e avaliação crítica. Arq. Esc. Med. Vet..

[11] Sbano J.C.N., Tsutsui J.M., Filho M.T., Junior W.M. (2004). The role of Doppler echocardiography in the evaluation of pulmonary hypertension. J. Bras. Pneumol..

[12] Chavatte-Palmer P., Remy D., Cordonnier N., Richard C., Issenman H., Laigre P., Heyman Y., Mialot J.P. (2004). Health status of cloned cattle at different ages. Cloning Stem Cells.

[13] Bowdle T.A. (2002). Complications of invasive monitoring. Anesthesiol. Clin. N. Am..

[14] Amory H., Linden A., Desmecht D., Rollin F., Genicot B., Lekeux P. (1991). Validation of the thermodilution techinique for the estimation of the cardiac output in the unsedated calf. J. Vet. Med. Ser. A.

[15] Keegan R.D., Greene S.A., Valdez R.A., Knowles D.K.l. (2006). Cardiovascular effects of desflurane in mechanically ventilated calves. Am. J. Vet. Res..

[16] Meirelles F.V., Birgel Junior E.H., Perecin F., Bertolini M., Traldi A.S., Pimentel J.R.V., Komninou E.R., Sangali J.R., Fantinato Neto P., Nunes M.T. (2010). Delivery of cloned offspring: Experience in Zebu cattle (Bos indicus). Reprod. Fert. Develop..

[17] Tsunoda Y. (2002). KATO, Y. Recent progress and problems in animal cloning. Differentiation.

[18] Birgel Junior E.H., Meirelles F.V., Komninou E.R., Nunes M.T., Pogliani F.C., Fantinato Neto P., Yasuoka M.M., Pimental J.R.V., Kubrusly F.S., Miglino M.A. (2011). Distúrbios clínicos observados nos primeiros 30 dias de vida de bezerros clonados da raça Nelore. Acta Sci. Vet..

[19] Hill J.R., Rousell A.J., Cibelli J.B. (1999). Clinical and pathologic features of cloned transgenic calves and fetuses (13 case studies). Theriogenology.

[20] Fatinato Neto P. (2015). Pulmonary Function Tests to Evaluate Gas Exchange and Pulmonary Capacity in Neonatal Cattle. Ph.D. Thesis.

[21] Amory H., Desmecht D., Linden A., McEntee K., Rollin F., Genicot B., Lekeux P. (1993). Growth-induced haemodynamic changes in healthy Friesian calves. Vet. Rec..

[22] Shellenberger N.W., Collinsworth K.K., Subbiah S., Klein D., Neary J.M. (2020). Hypoxia induces an increase in intestinal permeability and pulmonary arterial pressures in neonatal Holstein calves despite feeding the flavonoid rutin. J. Dairy Sci..

[23] Linden A., Desmecht D., Amory H., Lekeux P. (1999). Cardiovascular response to intravenous administration of 5-hydroxytryptamine after type-2 receptor blockade, by metrenperone, in healthy calves. Vet. J..

[24] Neary J.M., Garry F.B., Holt T.N., Knight A.P., Gould D.H., Dargatz D.A. (2013). Pulmonary arterial pressures, arterial blood-gas tensions, and serum biochemistry of beef calves born and raised at high altitude. Open Access Anim. Physiol..

[25] Shirley K.L., Beckman D.W., Garrick D.J. (2008). Inheritance of pulmonary arterial pressure in Angus cattle and its correlation with growth. J. Anim. Sci..

[26] Bleul U., Bircher B., Jud R.S., Kutte A.P.N. (2010). Respiratory and cardiovascular effects of doxapram and theophylline for the treatment of asphyxia in neonatal calves. Theriogenology.

[27] Amory H., Linden A.S., Desmecht D.J.M., Rollin F.A., McEntee K., Lekeux P.M. (1992). Technical and methodological requirements for reliable haemodynamic measurements in the unsedated calf. Vet. Res. Commun..

[28] Batchelder C.A., Whitcomb M.B., Famula T.R., Rodriguez-Villamil P., Bertolini M., Hoffert-Goeres K.A., Anderson G.B. (2017). Cardiac adaptations in SCNT newborn cloned calves during the first month of life assessed by echocardiography. Theriogenology.

[29] Davidson C., Bonow R.O., Bonow R.O., Mann D.L., Zipes D.P., Libby P. (2012). Cardiac Catheterization. Braunwald’s Heart Disease: A Textbook of Cardiovascular Medicine.

[30] Pogliani F.C. (2010). Parâmetros Ecodopplercardiográficos em Bezerros da Raça Nelore Originados Através de Transferência Nuclear de Células Somáticas Adultas—Clonagem. Ph.D. Thesis.

[31] Sidi D., Kuipers J.R.G., Heymann M.A., Rudolph A.M. (1983). Effects of ambient temperature on oxygen consumption and the circulation in newborn Iambs at rest and during hypoxemia. Pediatr. Res..

[32] Agata Y., Hiraishi S., Oguchi K., Misawa H., Horiguchi Y., Fujino N., Kimio Yashiro K., Shimada N. (1991). Changes in left ventricular output from fetal to early neonatal life. J. Pediatr..

[33] Teitel D.F., Iwamoto H.S., Rudolph A.M. (1990). Changes in the pulmonary circulation during birth-related events. Pediatr. Res..

[34] Feitosa F.L.F., Perri S.H.V., Bovino F., Mendes L.C.N., Peiró J.R., Gasparelli E.R.F., Yanaka R., Camargo D.G. (2011). Evaluation of the vitality of nelore calves born after normal or dystocic parturitions. Ars. Vet..

[35] Piccione G., Casella S., Pennisi P., Giannetto C., Costa A., Caola G. (2010). Monitoring of physiological and blood parameters during perinatal and neonatal period in calves. Arq. Bras. Med. Vet. Zootec..

[36] Bleul U., Lejeune S., Schwantag S., Kahn W. (2007). Blood gas and acid-base analysis of arterial blood in 57 newborn calves. Vet. Rec..

[37] Mess A.M., Ferner K.J. (2010). Evolution and development of gas exchange structures in Mammalia: The placenta and the lung. Respir. Physiol. Neurobiol..

[38] Paiano R.P. (2018). Effects of Anemia on Periparturient Cows. Master’s Dissertation.

